# Disparities in referrals to fertility preservation services for a pediatric and adolescent population

**DOI:** 10.1007/s10815-025-03581-8

**Published:** 2025-07-17

**Authors:** Erin Isaacson, Susan J. Woolford, Cheyney Dobson, Harlan McCaffery, Monica W. Rosen

**Affiliations:** 1 Department of Obstetrics and Gynecology, University of Michigan, Ann Arbor, MI USA; 2 Department of Pediatrics, University of Michigan, Ann Arbor, MI USA; 3 University of Michigan Child Health Evaluation and Research Center, Ann Arbor, MI USA

**Keywords:** Fertility preservation, Equitable care, Language preference, Healthcare disparities

## Abstract

**Purpose:**

This study aimed to determine if clinicians who provide potential gonadotoxic medications refer pediatric patients differently for a fertility preservation (FP) consultation based on race, ethnicity, socioeconomic status, or preferred language of the patient.

**Methods:**

This was a retrospective cohort study at a single tertiary care center. Patients assigned female at birth aged 5–21 years who underwent treatments considered medium to high risk for gonadotoxicity from 2017 to 2022 were included. Patients were excluded if they were referred from outside institutions already undergoing treatment or if they had a diagnosis of hemophagocytic lymphohistiocytosis. The primary outcome in the study was the presence of a referral to the FP team for patients undergoing gonadotoxic therapy.

**Results:**

The total cohort included 236 patients, 122 (51.6%) of whom were referred to the fertility preservation team. There was no significant difference in patients’ racial or ethnic backgrounds between referred and not referred. Those not referred were significantly younger (13.4 vs 16.3, *P* < .001) and less likely to speak English (7.1% vs .8%, *P* = .013). Patients less than 10 years of age made up 36.8% of those not referred, and only 1.6% of those referred. There was no difference in average Area Deprivation Index score between those referred and not (4.4 vs 5.0, *P* = .16), and referrals did not differ significantly between patients of low, medium, or high socioeconomic status (*P* = .28).

**Conclusion:**

Only half of appropriate patients were referred for fertility preservation counseling, and non-English speakers were significantly less likely to be referred, demonstrating a substantial need to address underlying referral disparities.

## Introduction

Fertility preservation (FP) is considered a key component of survivorship planning and is important for clinicians to discuss with patients prior to their undergoing gonadotoxic therapy. Over 20,000 pediatric and reproductive-aged patients are treated with chemotherapy and/or radiation annually for cancer and other conditions, and there is an upward trend in the survivorship annually due to medical advances [1–3]. Though gonadotoxicity risk differs significantly between patients due to factors such as age and dose and duration of treatment, female childhood cancer survivors have high documented rates of infertility (11–40%) and primary ovarian insufficiency (8%) [4–6]. Given these risks, multiple professional societies, including the American Society for Reproductive Medicine (ASRM) and the American Society of Clinical Oncology (ASCO), have published recommendations emphasizing the importance of early, robust FP counseling and intervention [7, 8]. While disparities in accessing FP resources among adults have been well-documented, data are limited for the pediatric and adolescent and young adult (AYA) populations. Even so, the data that is available suggests the presence of inequities [9–11]. Recent studies have found that AYA patients of a minority race or ethnicity are significantly less likely to receive FP when desired, but there is a paucity of literature examining inequities in initial referral for FP counseling [12]. Therefore, this study aimed to determine if clinicians administering potentially gonadotoxic therapies refer pediatric and AYA patients to FP consultation differently by age, race, ethnicity, socioeconomic status, or primary language spoken.

## Material and methods

This was a retrospective cohort study at a single academic medical center (serving as a tertiary referral center for oncology care) of patients assigned female at birth aged 5–21 years. Patients were included if they underwent radiology or chemotherapy treatment considered medium or high-risk for gonadotoxicity from January 2017 to December 2022. Treatments meeting criteria were determined primarily from the risk stratification system published by Meacham et al. [13], but also confirmed in conjunction with hematology-oncology and reproductive endocrinology specialists.

We performed an electronic health record (EHR) search review using EPIC Clarity to identify our cohort. Patients who met age criteria within the study period were identified for inclusion if they received a physician ordered for a formulation of one of the following medications: cyclophosphamide, ifosfamide, chlorambucil, melphalan, busulfan, procarbazine, carboplatin, cisplatin, doxorubicin, lomustine, temozolomide, vinplastine, vincristine, 5-fluorouracil, 6-mercaptopurine, bleomycin, cytarabine, dactinomycin, daunorubicin, etoposide, gemcitabine, or idarubicin. Standard referral practice for fertility preservation at our institution includes all patients who are or will be undergoing the above treatments in plans that carry moderate to severe risk of gonadotoxicity.

Patients were manually excluded through chart review if they were referred from outside institutions or were already undergoing potentially gonadotoxic therapy. They were also excluded if they had an underlying diagnosis of hemophagocytic lymphohyistiocytosis, given the severity of this condition and high overall mortality rate [14]. Lastly, they were excluded if they received a single dose of a high-risk medication for an unrelated condition. For example, several patients with vascular malformations received a single dose of 15 mg IV bleomycin, which is unlikely to be gonadotoxic.

The primary outcome in the study was the presence of a referral to the FP oncofertility team for patients undergoing gonadotoxic therapy. The patients meeting inclusion criteria were compared to the FP oncofertility team’s referral database to determine if a referral occurred.

The primary exposures were race, ethnicity, preferred language, and age. Potential confounders included age and socioeconomic status as estimated by the Area Deprivation Index (ADI), a measure of neighborhood deprivation developed by the University of Wisconsin Center for Health Disparities Research [15]. Patients were considered prepubertal if they were aged < 10 years.

Chart abstraction was performed by the study authors. Demographic information including age, race, ethnicity, preferred language, and address was collected from available information in the EHR. We mapped ADI state decile rankings to each patient by census block group using patient addresses recorded in the EHR at the time of the medication order. Patients could receive a score from 1 to 10, with 10 representing the greatest deprivation.

Patients were categorized by three racial groupings as self-reported in the EHR that allowed for maximizing statistical predictive power: Black or African American (any designation in a patient’s record), Other (any designation of Asian, Native Hawaiian/Pacific Islander, Native American/Alaskan Indian, Middle Eastern/North African, Other racial category, or Not specified/Did not disclose and no designation of Black or African American in a patient’s record), or White (as the sole designation in a patient’s record). Patients were additionally categorized by ethnicity (Hispanic or non-Hispanic) and primary language spoken (English or non-English).

Statistical analysis was performed using R version 4.4.2 (R Foundation for Statistical Computing, Vienna, Austria). Patients who had referrals for fertility preservation consults were compared to those who did not by race, ethnicity, preferred language, age, and ADI using Fisher’s exact test and Student’s *t*-test. We then used logistic regression to model the odds of fertility preservation consult with the above independent variables. This study was deemed exempt from review by the University of Michigan Medical School Institutional Review Board.

## Results

The total cohort included 236 patients. Of those, 122 (51.6%) received a referral for FP consultation. Most patients in the total cohort were White (73.7%) and Non-Hispanic (92.8%). There were no significant differences in patients’ racial or ethnic backgrounds for those who did or did not receive a referral (Table 1). Most patients were English-speaking (96.2%), but the nine patients who did not speak English as their preferred language were significantly less likely to receive a FP team referral (*P* = 0.032). These patients primarily spoke Spanish, Arabic, and Albanian.

**Table 1:** Patient demographics by fertility preservation referral status

Characteristic	Not referred	Referred	*P* value
Race			.082
White	87 (76.3)	87 (71.3)	
Black	17 (14.9)	30 (24.6)	
Other^a^	10 (8.8)	5 (4.1)	
Native American/Alaskan Indian	0	1	
Not specified/Did not disclose	5	2	
Middle Eastern/North African	2	2	
Asian	5	0	
Ethnicity			.701
Non-Hispanic	104 (91.2)	115 (94.2)	
Hispanic	10 (8.8)	7 (5.8)	
Preferred language			.07
Albanian	2 (1.8)	0 (0.0)	
Arabic	2 (1.8)	1 (0.8)	
English	106 (93.0)	121 (99.2)	
Spanish	4 (3.5)	0 (0.0)	
Preferred language (English vs non)			.032
English	106 (93.0)	121 (99.2)	
Non-English	8 (7.0)	1 (0.8)	
Age, years^b^	13.4 ± 5.3	16.3 ± 3.2	<.001
Mean ADI score^b^	5.0 ± 2.9	4.4 ± 2.9	.159
Socioeconomic status^c^			.285
Low	42 (36.8)	51 (42.1)	
Medium	33 (28.9)	40 (33.1)	
High	39 (34.2)	30 (24.8)	

Data presented as *n* (%) except where noted

*ADI* Area Deprivation Index

^a^Any designation of Asian, Native Hawaiian/Pacific Islander, Native American/Alaskan Indian, Middle Eastern/North African, Other racial category, or Not specified/Did not disclose and no designation of Black or African American in a patient’s record

^b^Mean ± SD

^c^Estimated by the Area Deprivation Index

Older patients were more likely to be referred for FP counseling (mean age 16.3 ± 3.2 years vs 13.4 ± 5.3 years, *P* < 0.001). The mean ADI score did not differ significantly between groups, as patients came equally from areas of low, medium, and high socioeconomic status (Table 1).

On linear regression, odds ratios were calculated as the ratio of the odds of patients receiving a referral in comparison to the reference categories (Table 2). Black patients had slightly higher odds compared to White patients (OR 1.94; 95% CI 0.92–4.2; *P* = 0.087), but this was not statistically significant (Table 2). Patients whose primary language was non-English had lower odds of receiving a referral, but this also did not reach statistical significance (OR 0.22; 95% CI 0.01–1.49; *P* = 0.187). Increasing age of patients led to significantly higher odds of receiving a referral (OR 1.16; 95% CI 1.09–1.24; *P* < 0.001) (Table 2).

**Table 2:** Odds ratios of receiving a fertility preservation referral

Characteristic	OR	95% CI	*P* value
Race			
White	Ref	Ref	
Black	1.94	0.92–4.2	.087
Other^a^	0.81	0.23–2.78	.731
Ethnicity			
Non-Hispanic	Ref	Ref	
Hispanic	1.01	0.31–3.35	.988
Preferred language			
English	Ref	Ref	
Non-English	0.22	0.01–1.49	.187
Age	1.16	1.09–1.24	.000

^a^Any designation of Asian, Native Hawaiian/Pacific Islander, Native American/Alaskan Indian, Middle Eastern/North African, Other racial category, or Not specified/Did not disclose and no designation of Black or African American in a patient’s record

## Discussion

The purpose of this study was to examine if clinicians administering gonadotoxic therapies refer patients to FP counseling differently by age, race, ethnicity, socioeconomic status (determined by ADI), or primary language spoken. We found that patients who spoke a primary language other than English were less likely to receive a referral for FP counseling compared to their English-speaking peers. Age was also a significant factor in referral patterns, as younger patients were significantly less likely to be provided with a referral for FP counseling. We did not see any disparities in referral to FP services for those of minority race or ethnicity or for patients who lived in disadvantaged neighborhoods of lower socioeconomic status.

The finding that English speakers are more likely to be referred for FP counseling than non-English speakers is rarely addressed in current literature. Few studies mention the challenges of a language barrier on accessing this area of medical care, although this bears important significance when considering the trend toward differences by race and ethnicity [10–12, 16, 17].

A recent international systematic review on barriers to fertility care briefly addressed language along with lack of fertility-related knowledge and found that low health literacy about fertility was often directly related to language barriers [17]. Lack of availability of appropriate interpreters in a timely fashion may dissuade providers from addressing complex issues, or they may resort to rudimentary communication with informal interpreters or gestures, leading to incomplete counseling or significant gaps in knowledge [17–19].

Another recent study looked at inequity of fertility treatment for non-English speaking patients, finding that these patients had higher all-cause treatment cancelation rates, lower in vitro fertilization cycles, and longer duration from first appointment to starting treatment [20]. Continued research on this rarely mentioned disparity, especially in an AYA population, is important for ensuring equitable access to FP counseling and services.

Our study also found that younger patients were significantly less likely to be referred for FP counseling. It is important to note that our institution does not currently offer ovarian tissue cryopreservation (OTC), which is the only available FP option for prepubertal patients. It is possible that this influenced a lack of referrals in this cohort. However, according to ASRM and ASCO guidelines, even if OTC is not offered at a provider’s institution, patients and families should still be counseled on all options that could be available elsewhere so they can make an informed choice about how to proceed [8, 21, 22].

In 2018, ASRM removed the experimental label on OTC [21, 23], but barriers to accessing it continue to exist for AYA populations, including a lack of available centers and variations in provider knowledge to assess risk for infertility and refer for counseling [24–26]. Addressing these barriers through continued provider education and institutional support of OTC programs—and FP in general—is crucial for delivering comprehensive, equitable care and counseling to all AYA at risk for infertility [27].

We did not see any disparities in referral to FP services for those of minority race or ethnicity or patients who lived in disadvantaged neighborhoods. Current literature notes significant disparities for racial minorities in accessing and utilizing FP resources in both adolescent and adult populations [9, 12, 16, 28]. However, there is no data specifically looking at referrals for counseling, especially for the AYA population. In contrast to current FP literature, our study did not find disparities in race or ethnicity. This may be due to our institution’s geographic area, where racial and ethnic minority populations remain small, resulting in a study population with limited diversity. Further studies are needed to specifically examine accessibility of adequate counseling for at-risk AYA populations.

Overall, our institution had an overall low rate of referral for FP counseling (51.6%) at the time of this study, despite the presence of an established FP program and coordinator. This is likely multifactorial; lack of prepubertal FP options at our institution may dissuade providers from referral and could also be a cause of a significant knowledge gap for providers who are unaware of OTC. It is also possible that patients and parents are not being given adequate information when an FP consult is offered or recommended, and may therefore decline prior to any contact with the FP team. Overall, there is significant work to be done to improve education and ensure FP counseling is appropriately described and offered to every at-risk patient.

The strengths of this study include its multi-year analysis and that the study institution is a tertiary referral center for oncology care. The presence of FP and navigation teams at our institution is also a strength, as it removes many potential barriers to FP counseling. Our demographic breakdown coincided with our hospital’s typical patient population, and patients were equally distributed in neighborhoods representing a wide range of socioeconomic status.

The results of this study should be considered within the context of certain limitations. It was a retrospective study that was unable to account for undocumented counseling by providers or patients who may have declined FP counseling. Furthermore, the small sample size of non-English-speaking and racial and ethnic minority patients likely hampered our ability to find statistical significance in regression models. Lastly, as mentioned previously, the lack of a local OTC program may have contributed to a lack of referrals for younger patients. Future development of strategies to improve referral patterns will be crucial to achieve equitable care, and could include continued education for clinical providers, infographics for patients and families in multiple languages, and expanded access to in person interpreter services.

## Conclusion

Many barriers continue to exist for pediatric and AYA patients undergoing potential gonadotoxic therapy to access FP counseling and resources, even in the setting of an established FP program. Language barriers and the effects on patients’ and caregivers’ understanding of infertility risk and FP options have not been well-studied. Determining strategies to address language barriers, and other disparities in access are essential to providing equitable FP care for individuals at risk of gonadotoxicity.

## Data Availability

The data that supports the findings of this study are not publicly accessible but can be available from the authors upon request.
